# Pilot production of a sensitive ELISA kit and an immunochromatographic strip for rapid detecting citrinin in fermented rice†

**DOI:** 10.1039/d2ra02823a

**Published:** 2022-07-08

**Authors:** Shih-Wei Wu, Jiunn-Liang Ko, Biing-Hui Liu, Feng-Yih Yu

**Affiliations:** Graduate Institute of Medicine, Chung Shan Medical University Taichung 40201 Taiwan; Graduate Institute of Toxicology, College of Medicine, National Taiwan University Taipei 10051 Taiwan biingliu@ntu.edu.tw +886-2-23123456-88602; Department of Medical Research, Chung Shan Medical University Hospital Taichung 40201 Taiwan; Department of Biomedical Sciences, Chung Shan Medical University Taichung 40201 Taiwan fengyu@csmu.edu.tw +886-4-24730022-11816

## Abstract

Citrinin (CTN) is a mycotoxin primarily produced by *Monascus* species. Excess consumption of CTN may lead to nephrotoxicity and hepatotoxicity. A pilot study for commercial production of competitive direct enzyme-linked immunosorbent assay (cdELISA) kit and an immunochromatographic strip (immunostrip) for screening CTN in red yeast rice is established in this study. The coating antibody and the CTN–horse radish peroxidase (HRP) concentrations were optimized to increase the sensitivity and specificity of cdELISA kit. The conjugation methods/ratios of CTN to HRP as well as the long-term stability of kit components were also evaluated. The IC_50_ and detection limit of the ELISA kit were determined to be 4.1 and 0.2 ng mL^−1^, respectively. Analysis of 20 red yeast rice samples using ELISA kits revealed the contamination levels of CTN from 64 to 29 404 ng g^−1^. The on-site rapid detection of CTN with the immunostrip showed that CTN levels in seven samples exceeded the regulatory limit of 5 ppm. Additionally, the coefficient correlation between the results of HPLC and ELISA kits of 20 samples was 0.96. Sensitive and convenient tools at commercial levels for detection of CTN contamination in food are established herein to protect the health of the public.

## Abstract 2

Rapid detection of citrinin in red yeast fermented products with ELISA kit and immunostrip and confirmed with HPLC.

## Graphical abstract

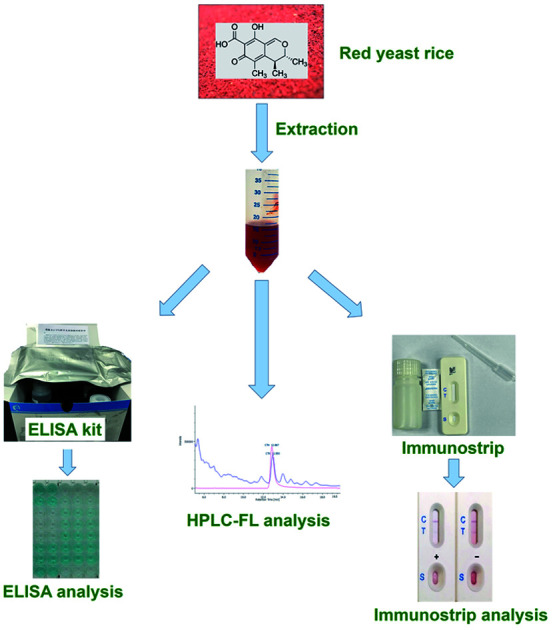

## Introduction

Red yeast rice, a *Monascus*-fermented product, has been widely used as health dietary supplements in many regions, such as Japan, Taiwan, China and East Asia. Red yeast fermented products are known to effectively reduce the cholesterol level, blood pressure and blood sugar in human beings, and they also demonstrate anti-inflammation, anti-aging and dementia prevention ability.^1,2^ However, citrinin (CTN), a small fungal secondary metabolite with a molecular weight around 250 Da (Fig. S1†), can also be bio-synthesized during the *Monascus* fermentation process.^3,4^*Monascus* samples collected in the Netherlands were all contaminated with CTN at concentrations ranging from 0.2 to 17.1 ppm.^5^ A survey by the Taiwan government also indicated that 69% of red yeast rice was contaminated with CTN, and the mean contamination level was 13.3 ppm.^6^ Excessive consumption of CTN may cause diseases and developmental toxicity of multiple organs, including the kidneys, liver, and intestines.^7–10^

Taiwan FDA has set a maximum allowable concentration of CTN in red yeast rice at 5 ppm (μg g^−1^). The government of Japan also sets a regulatory level for 0.2 ppm CTN in fermented products.^6^ To determine CTN contamination levels in red yeast rice, government officials typically apply high-performance liquid chromatography (HPLC) equipped with fluorescence detection (FL) and liquid chromatography with tandem mass spectrometry (LC/MS/MS).^11–14^ These chemical methods can accurately quantify CTN in samples, but expensive instruments, well-trained personnel, time-consuming process and difficult to implement outside the laboratory are obstacles to hinder the involvement of public in CTN detection.

Sensitive and convenient immunoassays can be used to analyze a large number of samples quickly and easily, without the aid of instruments. To measure the contamination levels of CTN in food and feed, highly sensitive ELISAs and an immunochromatographic strip (immunostrip) for on-site detection have been developed.^15–18^ The principle of direct competitive ELISA for CTN is to use the specific antibodies coated onto the plate. The CTN standard/samples and fixed amount of CTN–HRP were added to compete the limited antibodies in the well. Then, the enzyme substrate was added to the well for the color development. The higher free toxin gave less color and the levels of toxin in samples was quantified by the standard curve. The rapid immunostrip is a membrane-based immunoassay, in which stable gold nanoparticle (GNPs) conjugates are often used as a color probe.^19,20^ When the assay begins, the test samples mixed with antibody-gold nanoparticle (Ab-GNPs) conjugates will flow by capillary action onto the nitrocellulose (NC) membrane where they interact with the test and the control lines. The detecting results of the immunostrip is obtained within just 10 min and is easily operated by un-trained personnel. However, an immunostrip for detecting CTN contamination is not commercially available so far.

Although we have developed ELISA and immunostrip for CTN in our previous studies.^18^ To generate sensitive and reliable commercial immunoassays for CTN detection, the primary goal of this study concentrates on optimizing many critical experimental conditions to make the ELISA kit and immunostrip kit meet the criteria of commercial products. During the pilot production process, we focused on evaluating and improving long-term stabilities of kit components, examining various conjugation methods and regent ratios for increasing CTN detection sensitivity, and also optimizing the CTN Ab-GNPs. Furthermore, HPLC was applied herein to evaluate the performance and specificity of ELISA kit and immunostrip kit for CTN quantification in red yeast rice samples. The commercialized ELISA kit and immunostrip presented herein may provide the general public a chance to access cheap, rapid and simple tools for preventing CTN intoxication and protecting their health.

## Experimental

### Materials

Carboxymethoxylanmine hemihydrochloride (CMO), 1,1′-carbonyldiimidazole (CDI), 1-ethyl-3-[3-dimethylaminopropyl]-carbodiimide (EDC), *N*-hydroxysuccinimide (NHS), sodium bicarbonate, 1,4-butanediol diglycidyl ether, surfactant were purchased from Sigma (St Louis, MO, USA). Formic acid was obtained from Merck (Darmstadt, Germany). Both 50 nm and 70 nm gold nanoparticle were purchased from Cytodiagnostics Inc. (Burlington, Canada). The HPLC equipment consisted of a Jasco PU-4180 HPLC pump, a FP-1520 intelligent fluorescence detector and an AS-4050 autosampler was from Jasco (Tokyo, Japan). The 250 × 4.6 mm i.d., 5 μm, Atlantis T3 C18 reverse phase column was obtained from Waters (Milford, USA). A 40 × 4.0 mm i.d., 5 μm, Lichrospher C18 guard column was obtained from Merck (Darmstadt, Germany). CTN standard (Fig. S1A†) and other reagents were the same as the previous report.^18^ All reagents for pilot production of ELISA kit and immunostrip are prepared in our laboratory and sent to the biotechnology company for final immunostrip assembling and vacuum packaging techniques. All of the organic solvents and other chemicals used were reagent grade or better. An immunostrip scan reader CHR-110R was purchased from Kaiwood technology (Tainan, Taiwan). The anti-CTN polyclonal antibody was produced from our previous report.^18^

### Conjugation of different CTN conjugates

The various CTN conjugates, including CTN–HRP, CTN–OVA and CTN–BSA, were coupled with conjugation molar ratios as 29 : 1, 21 : 1 and 32 : 1, respectively, by mannich reaction using formaldehyde. The procedure of conjugation was as the previous report.^18^ Other CTN–HRP conjugation methods were presented in the supplemental file. All CTN conjugates were stored at −20 °C until used.

### Components of the ELISA kit

The kit components are as following. (1) An ELISA microplate (12 of 8-well strip) was precoated with 100 μL CTN antibody solution (concentration of 1 : 1000 diluted in PBS) and blocked with 0.1% BSA and 0.5% sucrose in 0.01 M PBS. Then the solution was aspirated and the plate was vacuum-packaged in an aluminum bag. (2) CTN standard solution (0–500 ng mL; 6 vials); (3) CTN–HRP conjugation solution (1 vial; 2000 folds dilution in stabilized buffer containing 0.01 M PBS, 0.1% BSA, 0.5% sucrose, and 20 ppm proclin); (4) washing buffer (10×; 1 bottle 30 mL of PBS-Tween, pH 7.4); (5) enzyme substrate solution (TMB; 1 bottle 12 mL); (6) stop buffer (1 bottle; 12 mL of 1 N HCl).

### Development and stability testing for the ELISA kits

The wells of a microtiter plate were coated with CTN Ab (8 μg mL^−1^, 100 μL) which was incubated at 37 °C for 1 h. After the plate had been washed four times with PBS-Tween (0.05% Tween 20 in 0.01 M PBS), the blocking solution (170 μL of 0.1% BSA and 0.5% sucrose in 0.01 M PBS) was added into the wells and kept at 37 °C for 1 h. Next, discarded the blocking solution and air dried it at 37 °C for 30 min. Then the plate was vacuum-packaged and stored at 4 °C until used. The ELISA kit package was opened and warmed to room temperature during the assay, 50 μL of CTN standard with concentrations from 0.1–500 ng mL^−1^ together with 50 μL of CTN–HRP conjugates (2000-fold dilution with the enzyme stabilized buffer, 0.01 M PBS containing 0.1% BSA, 0.5% sucrose, and 20 ppm proclin) were added into well. The competitive step of the assay proceeded at 37 °C for 30 min. Then, the plate was washed with PBS-Tween. Next, 100 μL of TMB substrate solution was added and waited for the color development for 15 min, then 100 μL of 1 N HCl was added to stop the reaction. Vmax ELISA reader was used to determine the absorbance value at 450–650 nm. For the stability testing for the antibody coated plate, the plates, CTN standard solution and CTN–HRP solution were stored at 4 °C for 1 year and monitored the absorbance value and IC_50_ of the ELISA once a month.

### Construction of the immunostrip

Different sizes (15–40 nm) of gold nanoparticles (GNPs) were synthesized with different reducing agent concentrations.^21^ Blue (70 nm) and purple (50 nm) colors of gold nanoparticles were obtained from Cytodiagnostics Inc. (Burlington, Canada). The procedure of CTN Ab coupling to the GNPs as probes was referred from the previous report.^21^ The components of immunostrip consisted of sample pad, release pad, absorbent pad, and nitrocellulose (NC) membrane with control line and test line. The control and test lines on the NC membrane were adsorbed with 0.25 μL of goat anti-rabbit secondary antibody (2 mg mL^−1^) and 0.25 μL of CTN–OVA conjugate (4 mg mL^−1^), respectively. After adsorption, the NC membrane with a plastic backing plate was dried overnight at room temperature. The CTN Ab-GNPs was added to a release pad and air-dried for 30 min at 37 °C. The release pad was pasted on the plate by overlapping 4 mm with the NC membrane. The other assembly steps are the same as the previous report.^21^ The assay procedure can be completed within 10 min and the result was obtained by visual. An immunostrip scan reader detects the density of the red line color on the test line.

### Long-term stability testing for the immunostrip

The protocol of the long-term stability testing was carried out as following. The immunostrip, which drawn the test line and control line and assembly, was placed in a vacuum-packaged bag after being dried at room temperature for 24 h. The immunostrip was stored at room temperature up for 1 year and analyzed the sensitivity and signal strength of the test line and control line at 6 and 12 month.

### HPLC

The HPLC method was established for determining the CTN levels. The HPLC equipment consisted of a Jasco PU-4180 RHPLC pump, a FP-1520 intelligent fluorescence detector were used. An AS-4050 autosampler injection valve was used to inject the standard and sample automatically. All CTN standards which dissolved in methanol were passed through 0.45 μm filter before injecting. Of the samples, 20 μL was injected onto an Atlantis T3 C18 reverse-phase column (5 μm particle size, 4.0 mm × 250 cm, Waters) equipped with a Lichrospher C18 guard column (5 μm particle size, 4.0 mm × 4.0 mm, Merck) and operated at room temperature with a flow rate of 1 mL min^−1^. The mobile phase consisted of solvents (acetonitrile/water/formic acid, 50 : 50 : 0.1, v/v/v). The fluorescence was measured at 500 nm with an excitation wavelength of 330 nm during 20 min.^6^ The collected data were analyzed with Jasco ChromNAV 2.0 HPLC Software. A calibration curve was generated using CTN standards of 0.05, 0.1, 0.15, 0.2, 0.25, 0.5, 1, 1.5, 2 and 2.5 μg mL^−1^. The calibration curve was quantified with peak area.

### Sample extraction

The sample preparation method was as previous reports.^6^ Generally, 2 g of red yeast rice was homogenized through the homogenizer. Next, 8 mL of extraction solvent (methanol) was added to the homogenization samples in the 50 mL centrifuge tube (dilution factor 4). The samples were vortexed for 1 min and heated in the water bath at 70 °C for 30 min. After the water bathing, each sample was cooled down at room temperature and then filtered through a 0.45 μm filter directly into a vial. The extracts were further diluted with 25 folds (dilution factor 100) for immunostrip analysis. On the other hand, the extracts were diluted with appropriate folds within the linear portion of the standard curve for ELISA analysis. All sample extracts were diluted with the BSA–Tween-PBS (0.1% BSA and 0.05% Tween 20 in 0.01 M PBS).

### Analysis of CTN contamination in *Monascus* fermented rice

#### Analysis of CTN contamination in *Monascus* fermented rice samples by the ELISA kits

The ELISA kit package was opened and warmed to room temperature, 50 μL of red yeast rice sample solutions which diluted with the BSA–Tween-PBS or CTN standard with concentrations from 0.1–500 ng mL^−1^ and together with 50 μL of CTN–HRP conjugates solution were added into well. After the reaction was finished at 37 °C for 30 min and the plate was washed with Tween-PBS four times. Then, 100 μL of TMB substrate were added to the wells and waited for 15 min. Next, 100 μL of 1 N HCl was added to stop the reaction. Vmax ELISA reader was used to determine the absorbance value at 450–650 nm. CTN concentrations in sample extracts were determined from the calibration curve.

#### Detection of CTN contamination in *Monascus* fermented rice samples with the immunostrip

The 200 μL of sample extract solutions (dilution factor 100) were added to the sample zone of the immunostrip with two duplicates. Each sample solution was flowed through the test line and control line by the capillary action. After 10 min, the results were obtained visually or by a strip scanner reader (Kaiwood technology).

#### Determination of CTN contamination in *Monascus* fermented rice samples by HPLC

The 1 mL of sample extracts were passed through a low protein binding 0.22 μm filter to discard the particle in solution. Then, the extracts (20 μL) were injected by the auto sample injection valve and analyzed by the Jasco ChromNAV 2.0 HPLC system. CTN concentrations in sample extracts were determined from the calibration curve, using the peak area for quantitation.

### Data analysis

The samples and standards were tested in triplicate and the mean values were obtained from the absorbance of ELISA. Establishment of the standard curves using software and formula was as the previous report.^21^

## Results

### Optimization of ELISA parameters

**Fig. 1:**
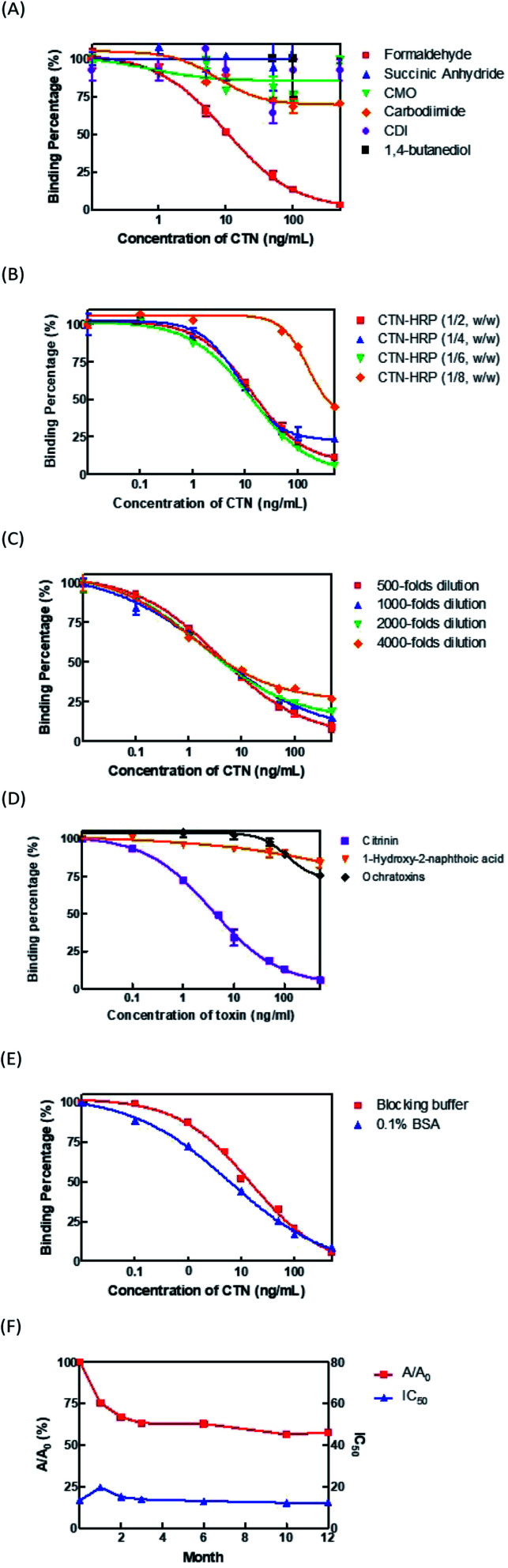
Characterization of the ELISA kit for CTN. (A) Comparison of the different conjugation method for CTN–HRP. (B) Comparison of the different conjugation ratio between CTN and HRP. (C) Comparison of CTN Ab concentration for coating onto the plate (D) cross-reactivities of CTN, 1-hydroxy-2-naphthoic acid and ochrartoxin A in the ELISA kit. (E) Comparison of the blocking buffer and 0.1% BSA using in the blocking step (F) stability analysis of the ELISA kit stability storage at 4 °C. *A* was the absorbance value of the D day; *A*_0_ was the absorbance value of the first day; the absorbance of the *A*_0_, CTN-free present was 2.5; each analysis was duplicated.

To optimize the developed ELISA, three aspects, the conjugation method of CTN–HRP conjugate, the conjugation ratio of CTN to HRP, and the dilution factor of the coating antibodies, are considered. First, the CTN was conjugated with HRP by the Mannich method using formaldehyde, succinic anhydride and CMO derivation followed by water-soluble carbodiimide (EDC), water-soluble carbodiimide (EDC), CDI, and 1,4-butanediol diglycidyl ether reagent. The sensitivity and absorbance values of the ELISA thus obtained were compared. The results revealed that the CTN–HRP conjugate that used formaldehyde was more effective than the conjugates with the other reagents, yielding the lowest IC_50_ of 6 ng mL^−1^ and the highest absorbance value of 1.8 (Fig. 1A). Different coupling ratios of CTN to HRP-1 : 2, 1 : 4, 1 : 6 and 1 : 8 (w/w) were used to test the sensitivity of the ELISA. In the Fig. 1B, CTN–HRP (1 : 8, w/w) yielded the worst IC_50_ of the ELISA whereas the other conjugates yielded the similar value of IC_50_. CTN–HRP conjugates with a coupling ratio of 1 : 6 (w/w) yielded the highest absorbance of 1.880. Therefore, CTN–HRP conjugates with conjugation ratio of 1 : 6 (w/w) was subsequently used. Next, the coated antibodies were diluted 500-, 1000-, 2000- and 4000-fold and the IC_50_ and maximal absorbance values of the ELISA were compared. The results revealed that no variation in the value of the IC_50_ among these groups, and the antibodies that were coated with dilutions of 500- and 1000-fold had higher absorbances of 1.147 and 1.006, respectively (Fig. 1C). Accordingly, the coated antibodies with 1000-fold dilution were used in the ELISA kit.

### Characterization of ELISA kit

Antibody (Ab) specific to CTN were used to develop in the ELISA kits. After the ELISA kit had been optimized, a CTN of 4.1 ng mL^−1^ was determined to cause 50% inhibition of CTN–HRP binding to the CTN antibodies (IC_50_). The detection limit (IC_10_) and the working scope (IC_20_ to IC_80_) were 0.2 ng mL^−1^ and 0.5–36.6 ng mL^−1^, respectively (Fig. 1D). Synthetic compound 1-hydroxy-2-naphthoic acid (Fig. S1B†) and ochrartoxin A (Fig. S1C†), both chemical structures similar to that of CTN, showed only 10% weak cross-reactivities with the CTN Ab at a level of 100 ng mL^−1^ (Fig. 1D).

### Analysis of long-term stability of ELISA kit

To test the long-term stability of ELISA kits in storage, stability analysis was conducted. A blocking buffer that contained 0.5% sucrose and 0.1% BSA were used to protect the CTN antibodies that were coated on the wells of the microplate. The results revealed that the IC_50_ of the blocking buffer group (14 ng mL^−1^) was higher than that of the 0.1% BSA group (6.0 ng mL^−1^) (Fig. 1E). The blocking buffer was used in the ELISA kits for protection during long-term storage. Fig. 1F presents the efficacy of an ELISA kit that was used once monthly otherwise stored at 4 °C. *A*/*A*_0_ (where *A* is the absorbance on day D, and the *A*_0_ is the absorbance on the first day) decreased to 75%, 67%, 63%, 62%, 56% and 55% at 1, 2, 3, 6, 10 and 12 month. The IC_50_ value changed only slightly.

### Optimization of immunostrip components

**Fig. 2:**
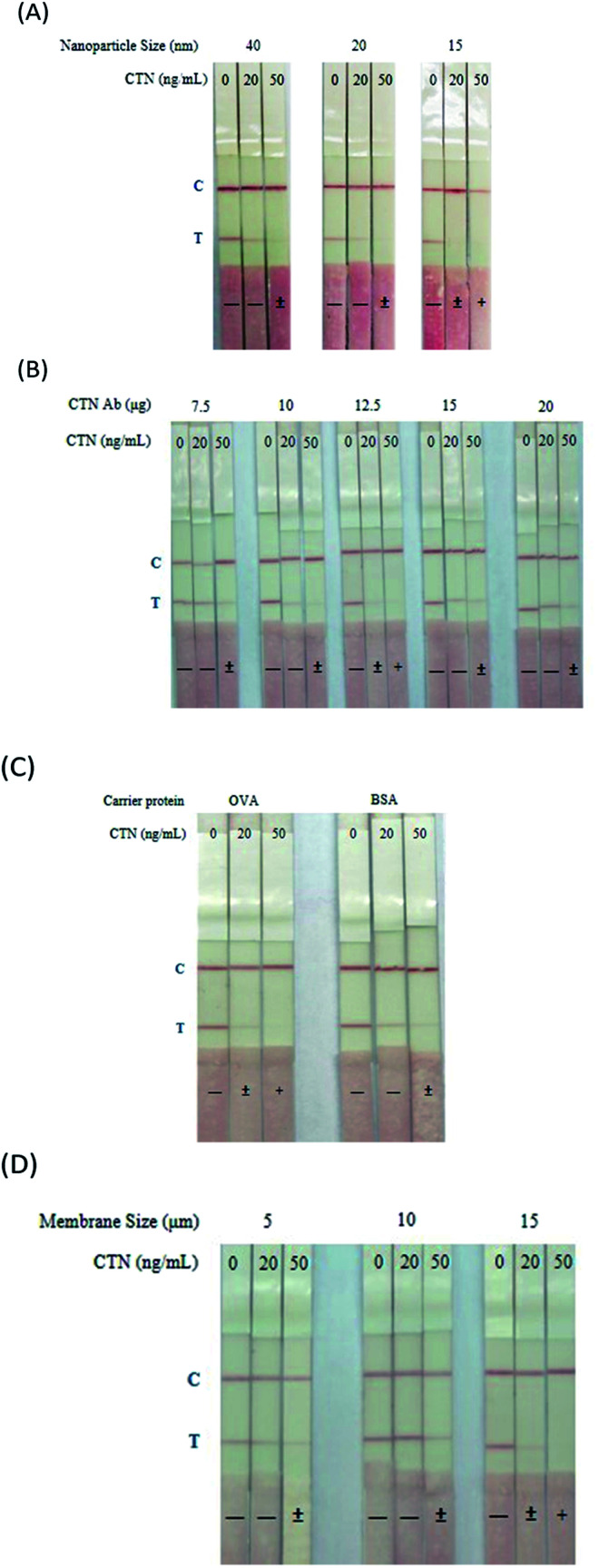
Optimization of the immunostrip for CTN. (A) Comparison of the particle size of the gold nanoparticle. (B) Comparison of the quantity of antibodies on the surfaces of gold nanoparticles. (C) Comparison of the test line reagents CTN–OVA and CTN–BSA. (D) Comparison of the pore size of nitrocellulose.

The immunostrip is a simple and rapid device for the on-site determination of CTN levels in red yeast rice samples. The CTN–OVA and secondary antibody are adsorbed onto the test line and control line, respectively. CTN occupies the CTN Ab binding sites on all of the CTN Ab-GNPs in the analysis solution when the CTN level exceeds a particular value. Hence, no free CTN Ab-GNPs bind with the CTN–OVA conjugate on the test line of the NC membrane. The absence of a red signal on the test line indicates that the sample is positive for CTN. When the CTN level is below the threshold, unsaturated CTN Ab binding sites binds to CTN–OVA conjugate on the test line. The two red signals that are therefore visible at the test line and the control line indicate that the sample is negative for CTN. To optimize the immunostrip, four parameters are considered; they are size of the gold nanoparticles, the quantity of antibodies on the surfaces of gold nanoparticles, the pore size of NC membrane and the amount of the carrier protein CTN–OVA on the test line. First, gold nanoparticles were synthesized with sizes of 15, 20 and 40 nm. Fig. 2A reveals that the 15 nm gold nanoparticle yielded a good signal strength on the test line and gave the best detection limit of 20 ng mL^−1^. Next, the quantities of antibodies that were adsorbed onto the surface of the gold nanoparticles surface were determined using 7.5, 10, 12.5, 15 and 20 μg of those antibodies. When 12.5 μg of antibodies was adsorbed onto the surface of the gold nanoparticles, not only was the test line strongest but also the production cost was lowest (Fig. 2B). CTN–OVA and CTN–BSA were then compared as test line reagents. As shown in Fig. 2C, CTN–OVA gave a better detection limit than CTN–BSA. Finally, the detection limit with different pore sizes of the NC membrane-, 5, 10 and 15.

### Characterization of immunostrip

**Fig. 3:**
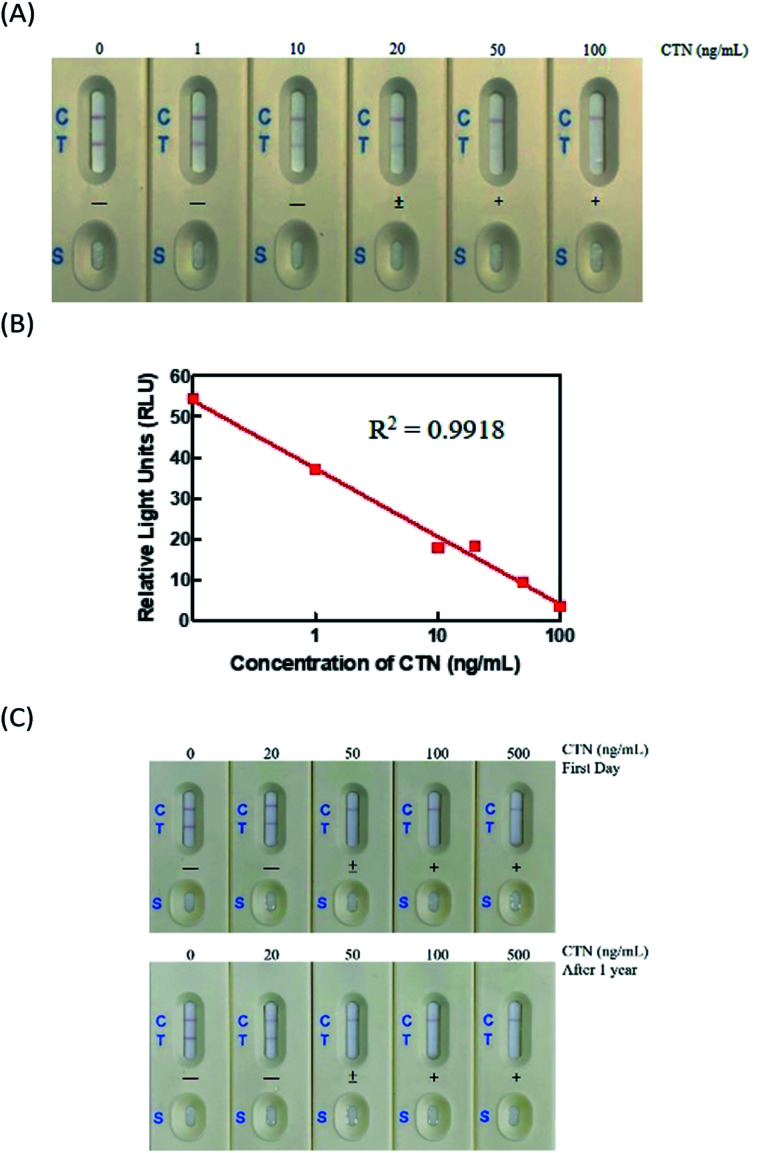
Characterization of immunostrip for CTN standard. (A) Visual detection limit of the immunostrip for CTN (B) the standard curve of the test line intensity on the immunostrip. (C) Long-term stability analysis of the immunostrip.

The immunostrip was exposed to CTN standard concentrations that prepared from a certified solution of CTN (0, 1, 10, 20, 50 and 100 ng mL^−1^). In Fig. 3A, 200 μL of each CTN standards were applied to the sample zone to initiate capillary motion. CTN at 50 or 100 ng mL^−1^ did not yield a red line on the test line, indicating that the detection limit of CTN of the immunostrip was between 20-50 ng mL; while the color density of the T line was determined by the strip scan reader and the value less than 15 RLU (relative light units) (Fig. 3B).

### Analysis of long-term stability of immunostrip

For the commercial production of immunostrips, their storage stability of immunostrip is important. Accordingly, a vacuum-packaged assembled immunostrip was stored at room temperature and the sensitivity and intensity of the test line were checked at 6 and 12 months. Visual inspection revealed that the strength of the color of the test line declined slightly for the CTN-free group but the detection limit of the immunostrip did not change through one year of storage (Fig. 3C). In addition, the intensities of their control lines also did not change. Therefore, the immunostrip that developed in this study can be stored for at least one year.

### Extensive analysis of *Monascus* fermented rice samples using ELISA kits, immunostrip and HPLC

#### Analysis of CTN contamination levels in *Monascus* fermented rice samples using ELISA kit

The ELISA kits were used to determine CTN contamination levels in 20 rice samples from local markets. Owing to the influence of the matrix, each sample extract was diluted with 0.1% BSA–Tween-PBS before analysis. The ELISA results indicated that all 20 rice samples were contaminated with CTN at concentrations from 64 to 29 404 ng g^−1^ (Table 1). Thus, the CTN contamination incidence rate was 100% and the concentrations exceeded the 5 ppm regulatory limit that had been set by the TFDA is 15% of the samples.

**Table 1:** Analysis of red yeast rice samples using ELISA kits, immunostrip and HPLC

Sample no.	ELISA kit (ng g^−1^)	Immunostrip^a^	HPLC (ng g^−1^)
1	1862 ± 372	—	1975
2	8577 ± 738	+	7417
3	2666 ± 260	—	3083
4	1539 ± 363	—	1756
5	156 ± 19	—	N.D.
6	29 404 ± 2433	+	4202
7	4195 ± 654	+	5551
8	4865 ± 495	+	4179
9	988 ± 197	—	984
10	483 ± 0	—	645
11	608 ± 24	—	567
12	64 ± 12	—	N.D.
13	2388 ± 189	—	929
14	1384 ± 116	—	1066
15	4012 ± 199	—	3993
16	4068 ± 93	+	4881
17	3390 ± 658	+	4669
18	487 ± 0	—	432
19	906 ± 0	—	860
20	14 158 ± 0	+	15 894

a Each sample extract was diluted 100 folds for immunostrip analysis to comply with TFDA regulation of 5 ppm.

#### Determination of CTN contamination levels in *Monascus* fermented rice samples using immunostrip

**Fig. 4:**
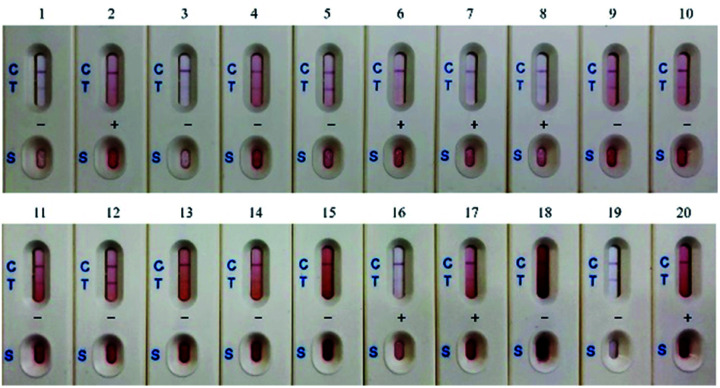
Analysis of CTN contamination levels in 20 red yeast rice samples with immunostrip.

Immunostrips were used to determine the CTN levels in the 20 rice samples. The rice extract solution (with a dilution factor of 100) was added to the wells of the immunostrip to determine whether the CTN contamination of samples exceeded the legal limit (5 ppm) in Taiwan. Table 1 shows that this limit of 5 ppm, set by the TFDA, was exceeded in samples 2, 6, 7, 8, 16, 17, and 20 (positive) and not in 13 samples (negative). In the Fig. 4, two clear red lines (C and T) correspond to a negative result and the red control line only corresponds to a positive result.

#### Determination of CTN contamination levels in *Monascus* fermented rice samples using HPLC

**Fig. 5:**
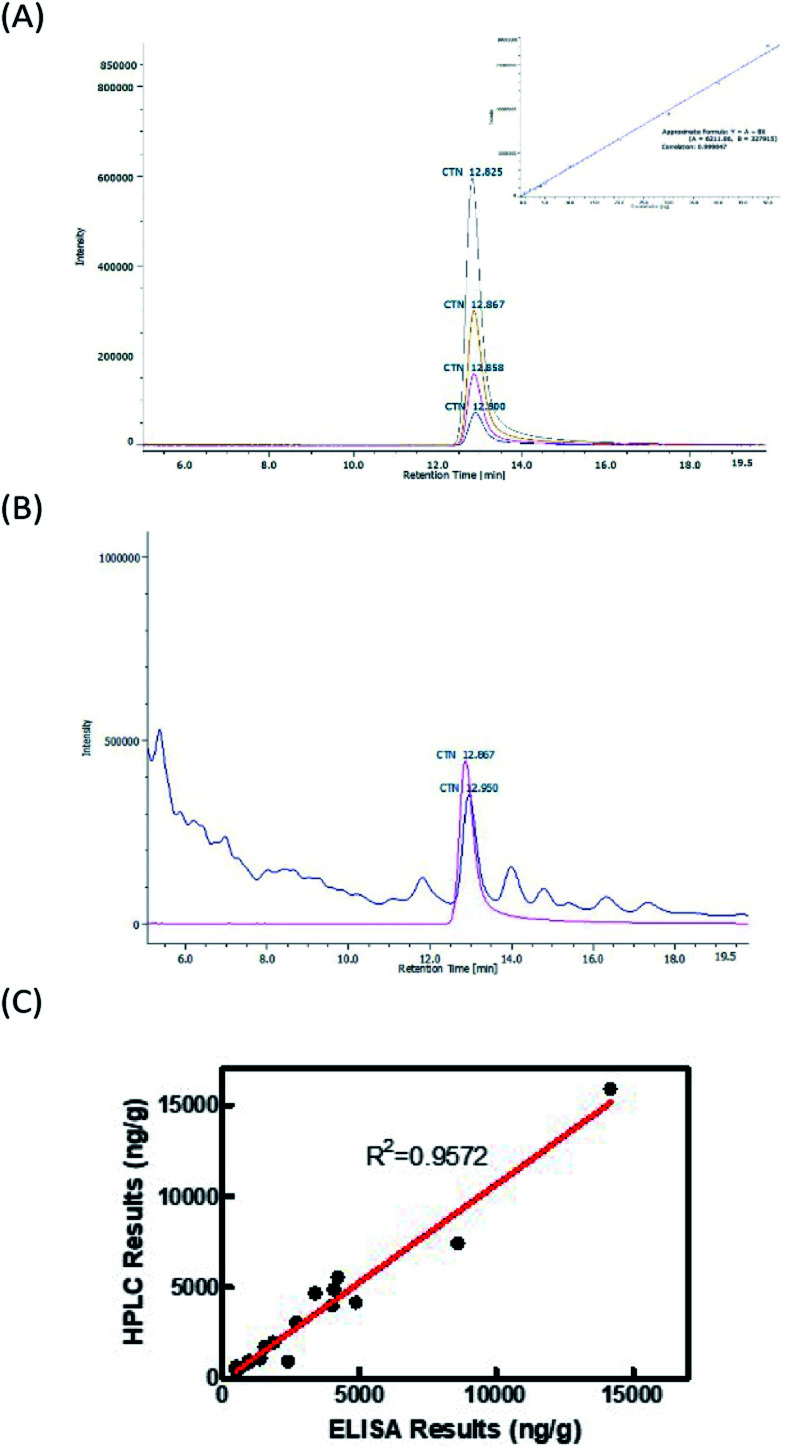
Analysis of CTN with high performance liquid chromatography (A) HPLC chromato-grams of 5 (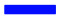), 10 (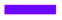), 20 (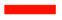), and 40 (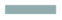) ng of CTN standard. The inlet figure is the calibration curve of CTN. (B) HPLC chromatograms of 30 ng of the CTN standard (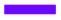) and one red yeast rice sample with CTN contamination (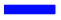) (C) the correlation coefficients of HPLC and ELISA kit for analyzing the CTN levels in red yeast rice samples.

The CTN standards (0.05–2.5 μg mL^−1^) were analyzed using an HPLC-FL method to establish a standard curve for calibration. Fig. 5A displays HPLC chromatograms of 5, 10, 20 and 40 ng CTN standards. These standards were well-identified with a retention time of 12.8–12.9 min (Fig. 5A) and the calibration curve was plotted in the inset. The correlation coefficient from the calibration curve was 0.999 and the detection limit of HPLC was 1 ng (insert in Fig. 5A). The HPLC method was further used to analyze the rice samples, confirming the results of ELISA kit and immunostrip. Table 1 presents the HPLC results. The contamination levels ranged from 432 to 15 894 ng g^−1^. The levels in samples 2, 7, and 20 were exceeded the regulatory limit of 5 ppm. Fig. 5B displays HPLC chromatograms of 30 ng of the CTN standard and one rice sample with CTN contamination. However, the contamination in samples 2 and 5 was below the HPLC detection limit. Fig. 5C showed that the coefficient correlation between the results of HPLC and ELISA kits was 0.957216. This correlation coefficient was obtained without considering sample 6 because the HPLC and ELISA results for this sample were different. Sample 6 contained many particles after the sample extraction procedure, possibly explaining the strong positive ELISA result.

## Discussion

Numerous people in Taiwan and East Asia consume the *Monascus* fermented rice for the benefit of their health benefit and for the purpose of food processing.^1,2^ In this study, 20 *Monascus* fermented rice samples were obtained from local markets and were found to have high rates of CTN contamination with incidences almost 100%; additionally, 20% of them exceeds the maximum allowable level of CTN in *Monascus*-fermented rice products, according to the regulation set by Taiwan government. Therefore, it is an urgent issue to provide easy and fast commercial tools for the public to aware the exposure levels of CTN in their diary supplements.

In our earlier report, a sensitive ELISA and immunostrips for CTN, based on laboratory scale, are initially developed. The IC_50_ and detection limit of CTN in the ELISA were determined to be 5 ng mL^−1^ and 0.2 ng mL^−1^, respectively.^18^ In order to improve the sensitivity of the ELISA kit for commercial production, this study first compared chemical coupling methods for the generation of CTN–HRP conjugates. A good coupling method may increase the coupling efficiency of CTN to HRP and also avoid the possibility to block the epitope structure on CTN.^22,23^ CTN is a small toxin with a molecular weight of 250 Da, so making the limited epitope of CTN available to antibody binding is important to promote the assay sensitivity. Among six different conjugation methods that have been examined (Fig. 1A), Mannich method using formaldehyde to react with several methyl groups (C9, C10, C11) on the left side of CTN was found to be the best coupling method to optimize the detection limit of ELISA kit (Fig. 1A). The general principle of water-soluble carbodiimide method is supposed to conjugate carboxylic group of compound to the amine group of target protein. However, the C12 carboxylic acid of CTN may interact with C8 hydroxyl group and C7 carbonyl group *via* hydrogen bonding.^15,24^ It prevents the successful application of carbodiimide method to link CTN with HRP. Similarly, the CDI method also demonstrated a poor coupling effect due to its intentionally reacting with the carboxylic group on C8 or C12 on CTN. The 1,4-butanediol diglycidyl ether, which conjugates the C7 carboxyl group of CTN with the amino group of HRP,^25^ cannot produce a sensitive product suitable for ELISA usage (Fig. 1A). Taken together, according to previous studies and our current experience, the function groups on C6 and C7 of CTN are probably the essential part of CTN epitope.^25,26^ Therefore, only the Mannich method that focuses on conjugating HRP to the left part (C9-11) of CTN can make the C6 and C7 (Fig. S1,† right part) available for antibody recognition.

This study further analyzed the various conjugation ratios of CTN to HRP to improve the sensitivity and absorbance value in commercial ELISA kits. Generally, the high ratio of HRP coupling to CTN may lead to a higher absorbance value, but the sensitivity would decrease simultaneously. We found that the best conjugation ratio of CTN to HRP, 1 : 6 by Mannich method, maximized the sensitivity of the ELISA kits (Fig. 1A and B). Additionally, the absorbance value using 1 : 6 CTN–HRP ratio was at least four times higher than those of CTN–HRP ratios at 1 : 2 and 1 : 4.

The ELISA developed at a laboratory scale generally cannot be stored at 4 °C for more than two weeks.^27^ To extend the shelf life of commercial ELISA kits, several critical factors involving in long-term storage were comprehensively evaluated. We found that addition of 0.5% carbohydrate into the BSA-PBS blocking solution can extend the shelf life of ELISA kit for up to one year without decreasing its sensitivity and absorbance values (Fig. 1E). The blocking step with 0.5% carbohydrate generates a film on the bottom of ELISA microplate to prevent the antibody from contacting air and then achieves the effect of increasing stability.

Further sealing microplates with industrial-level vacuum package by Advance Bio-pharmaceutical Inc. company also promotes the long-term stability of ELISA kits. Additionally, to keep CTN–HRP conjugation solution stable in kit for more than one year at 4 °C, the stabilized buffer which containing antibacterial agents, preservatives and an appropriate amount of BSA was used to replace simple PBS solution.

To establish a successful immunostrip for commercial usage, we optimized several critical parameters, including the size of gold nanoparticles (Fig. 2A), the amounts of antibody adsorbed onto gold nanoparticles (Fig. 2B), the carrier protein adsorbed onto the test line (Fig. 2C) and the pore size of NC membrane (Fig. 2D). In general, the nanoparticle with a larger size has a larger antibody absorption area which favors the strength of color signal on the test line of immunostrip.^28^ However, smaller particles yielding a slightly weaker signal on the immunostrip make the immunostrip to be more sensitive. Therefore, although some previous studies report the usage of 38–40 nm gold nanoparticle as color probe is suitable in immunostrip,^29^ we observed that 15 nm gold nanoparticle with red color demonstrated a better sensitivity and visual identification on the strip than 20 and 40 nm particles (Fig. 2A). On the other hand, 50 nm gold nanoparticle with purple color and 70 nm gold nanoparticle with blue color, obtained from Cytodiagnostics Inc., were also used in conjugating to Ab specific to CTN for examining their sensitivity in immunostrip (Fig. S2A†). Unfortunately, both nanoparticle probes with larger size and color other than red could not enhance the detection sensitivity and color intensity on the immunostrip (Fig. S2†).

In order to reach the goals of streaming the using steps of commercial immunostrip, a dry-type competition procedure was established in the current study. During the immunostrip assembling, gold nanoparticle-Ab conjugates were coated on the release pad on the NC membrane first, so the conjugates on the pad can catch the CTN in sample moving up by capillary flow from the sample area. The dry-type competition is better than the wet-type competition, in which a sample solution has to pre-mixed with gold nanoparticle-Ab conjugates in tube before being loaded into the sample area on immunostrip. The wet-type competition technique is commonly used in many experimental groups^30^ and also developed in our previous published paper;^18^ however, it is not convenient for immunostrip commercialization.

The results from ELISA kits showed that all of the *Monascus* fermented rice samples collected from local markets were contaminated with CTN. Additionally, the immunostrip revealed 7 out of 20 samples exceeds the regulatory level set by Taiwan FDA (Table 1). Similar phenomena were observed in Malaysia; all of 50 *Monascus* fermented rice samples at consumer level are contaminated with CTN ranging from 230–20 650 ng g^−1^.^31^ It suggests that CTN contamination in *Monascus* fermented rice is a big health issue in Asia. ELISA results were confirmed with HPLC-FL to show a correlation coefficient of 0.96 (Fig. 5); only the CTN levels in sample 6 were dramatically different between ELISA and HPLC. The extraction solution of sample 6 contained many particles, which settled on the bottom of microtiter plate to interfere the following colorimetric step. According to Table 2, the sensitivity of our CTN ELISA kit is better than those of commercial ELISA kits from four different companies. The validation of CTN ELISA kit analytical method with HPLC in a target concentration of 5 ppm was presented in the Table S1;† the specificity and sensitivity for CTN ELISA kit were 94% and 67%, respectively. The validation of CTN immunostrip screening method with HPLC in a target concentration of 5 ppm was presented in the Table S2;† the specificity and sensitivity for CTN immunostrip was 76% and 100%, respectively.

**Table 2:** Comparison of commercial CTN ELISA kit

ELISA kit (vendor name)	IC_50_ (ng mL^−1^)	Detection limit (ng mL^−1^)
This study	4.1	0.2
Asurgreen analysis Co	—	—
R-Biopharm AG	—	15
Beacon analytical Systems, Inc.	10	<1
Glory Science Co., Ltd	>100	>100

## Conclusions

This study focuses on establishing a pilot production of commercialized ELISA kit and immunostrip. The available products of ELISA kit and immunoscrip will be useful and practical tools to protect and improve the health of people and their communities. Especially, no immunostrip specific to CTN is commercially available so far to our knowledge. The detection limit of ELISA kit established herein was calculated to be 0.2 ng mL^−1^, and the concentration that caused 50% inhibition of the binding of CTN–HRP to the CTN Ab was 4.1 ng mL^−1^. Additionally, the detection limit of immunostrip was 20–50 ng mL^−1^. Both established ELISA kit and immunostrip exhibited a long-term stability for up to one year after vacuum package at the industrial level. Analysis of CTN contamination in *Monascus* fermented rice samples with ELISA kit and immunostrip were highly consistent with the data obtained from HPLC. Providing sensitive and convenient commercial kits for the public to detect CTN contamination in food on site will benefit the health of whole community.

## Author contributions

Shih-Wei Wu: formal analysis, methodology. Jiunn-Liang Ko: conceptualization, supervision. Biing Hui Liu: conceptualization, investigation, writing-review and editing. Feng-Yih Yu: conceptualization, investigation, supervision, writing-original draft preparation.

## Funding

This research was funded by Ministry of Science and Technology, Taiwan (MOST 109-2320-B-040-002-MY3) and Taiwan Food and Drug Administration (107TFDA-A-108).

## Conflicts of interest

The authors declare no conflict of interest.

## Supplementary Material

RA-012-D2RA02823A-s001

## References

[cit1] Cicero A. F. G. (2018). Recenti Prog. Med..

[cit2] Xiong Z., Cao X., Wen Q., Chen Z., Cheng Z., Huang X., Zhang Y., Long C., Zhang Y., Huang Z. (2019). Food Chem. Toxicol..

[cit3] Bennett J. W., Klich M. (2003). Clin. Microbiol. Rev..

[cit4] Chu F. S. (1991). Mutat. Res..

[cit5] Sabater-Vilar M., Maas R. F., Fink-Gremmels J. (1999). Mutat. Res..

[cit6] Liao C. D., Chen Y. C., Lin H. Y., Chiueh L. C., Shih D. Y. C. (2014). Food Control.

[cit7] Kitchen D. N., Carlton W. W., Tuite J. (1977). Vet. Pathol..

[cit8] Kumar M., Dwivedi P., Sharma A. K., Singh N. D., Patil R. D. (2007). Mycopathologia.

[cit9] Singh N. D., Sharma A. K., Dwivedi P., Patil R. D., Kumar M. (2007). J. Appl. Toxicol..

[cit10] Wu T. S., Yang J. J., Yu F. Y., Liu B. H. (2013). Toxicol. Sci..

[cit11] Huertas-Pérez J. F., Arroyo-Manzanares N., García-Campaña A. M., Gámiz-Gracia L. (2015). Food Addit. Contam., Part A: Chem., Anal., Control, Exposure Risk Assess..

[cit12] Lee C. L., Wang J. J., Pan T. M. (2006). J. AOAC Int..

[cit13] Odhav B., Naicker V. (2002). Food Addit. Contam., Part A: Chem., Anal., Control, Exposure Risk Assess..

[cit14] Vail R. B., Homann M. J. (1990). J. Chromatogr. A.

[cit15] Abramson D., Usleber E., Martlbauer E. (2001). Methods Mol. Biol..

[cit16] Cheng H., Yang Y., Chen Y., Chen X., Cai Z., Du A. (2018). PLoS One.

[cit17] Kong D., Xie Z., Liu L., Song S., Kuang H. (2017). Food Agric. Immunol..

[cit18] Wu S. W., Yu Y. A., Liu B. H., Yu F. Y. (2018). Toxins.

[cit19] Page Faulk W., Malcolm Taylor G. (1971). Immunochemistry.

[cit20] Paek S. H., Lee S. H., Cho J. H., Kim Y. S. (2000). Methods.

[cit21] Wu S. W., Ko J. L., Liu B. H., Yu F. Y. (2020). Toxins.

[cit22] Burnens A., Demotz S., Corradin G., Binz H., Bosshard Hans R. (1987). Science.

[cit23] Dhungana S., Fessler M. B., Tomer K. B. (2009). Methods Mol. Biol..

[cit24] Schwenk E., Alexander G. J., Gold A. M., Stevens D. F. (1958). J. Biol. Chem..

[cit25] Li Y., Wang Y., Guo Y. (2012). Food Agric. Immunol..

[cit26] Wang Y. Y., Li Y. N., Guo Y. H. (2010). Prog. Biochem. Biophys..

[cit27] Wu S. W., Wang M. Y., Liu B. H., Yu F. Y. (2020). J. Food Saf..

[cit28] Li Y., Li J., Huang H., Jian D., Shan Y., Wang S., Liu F. (2021). Food Control.

[cit29] Lou S., Ye J. Y., Li K. Q., Wu A. (2012). Analyst.

[cit30] Molinelli A., Grossalber K., Führer M., Baumgartner S., Sulyok M., Krska R. (2008). J. Agric. Food Chem..

[cit31] Samsudin N. I. P., Abdullah N. (2013). Mycotoxin Res..

