# Supplementary material for: Pilot production of a sensitive ELISA kit and an immunochromatographic strip for rapid detecting citrinin in fermented rice
Source: RSC Adv. 2022 Jul 8;12(31):19981–9. doi: 10.1039/d2ra02823a (PMC9264126; doi:10.1039/d2ra02823a)
Supplement: RA-012-D2RA02823A-s001 [file RA-012-D2RA02823A-s001.pdf]

Electronic Supplementary Material (ESI) for RSC Advances.
This journal is © The Royal Society of Chemistry 2022

Pilot Production of a Sensitive ELISA kit and an Immunochromatographic Strip for Rapid Detecting Citrinin in Fermented Rice

Received 00th January 20xx,
Accepted 00th January 20xx

DOI: 10.1039/x0xx00000x

Shih-Wei Wu,[a] Jiunn-Liang Ko, [a] Biing-Hui Liu *[b] and Feng-Yih Yu *[c,d]

Supplementary Methods

Conjugation of differents CTN-HRP conjugates

Crosslinking of CTN-HRP by carbodiimide method

The crosslinking of CTN to HRP with conjugation molar ratio as 29:1 by the water-soluble carbodiimide method was as following. Briefly, 2 mg of EDC in 0.02 mL DMSO and 1.5 mg of NHS in 0.02 mL DMSO were added to 1 mg of CTN in 0.1 mL methanol with stirring at room temperature for 2 h. Next, 6 mg of HRP in 0.6 mL 0.1 M carbonate buffer (pH 9.6) was gradually added to the mixture with steady stirring for 16 h at room temperature. Finally, the product was dialyzed against 0.01 M PBS for 3 days.

Coupling of CTN-HRP by succinic anhydride and carbodiimide method

The succinic anhydride (2.5 mg of succinic anhydride in 0.6 mL pyridine) was dropwise added to 5 mg of CTN in 0.25 mL methanol with steady stirring at 37°C for 60 h. After the reaction, the reaction solvent was discarded by the rotary evaporators and stored at -20°C until used. Next, the CTN-SH solution (1 mg of CTN-SH in 0.1 mL DMSO), EDC solution (2 mg of EDC in 0.02 mL DMSO) and NHS solution (1.5 mg of NHS in 0.02 mL DMSO) were mixed together with continuous stirring at room temperature for 2 h. Then, the HRP solution (6 mg of HRP in 0.6 mL carbonate buffer, pH 9.6) was added to the reactant with steady stirring at room temperature for 16 h. Finally, the CTN-HRP with conjugation molar ratio as 29:1 was dialyzed against 0.01 M PBS for 3 days.

Conjugation of CTN-HRP by CMO and carbodiimide method

The procedure for coupling the CTN-CMO-HRP conjugates was as following. First, 10 mg of CTN and 20 mg CMO were dissolved in 3 mL and 6 mL reaction solution (pyridine/methanol/deionized water, 1/4/1, v/v/v), respectively. Both solutions were mixed evenly with steady stirring for 3 h at 37°C and another 16 h at room temperature. The reactant was discarded reaction solution by the rotary evaporators and stored at -20°C until used. Second, 1 mg of CTN-CMO in 0.1 mL DMSO and the EDC solution (2 mg of EDC in 0.02 mL DMSO) and NHS solution (1.5 mg of NHS in 0.02 mL DMSO) were mixed with stirring. Next, the 6 mg of HRP in 0.6 mL 0.1 M carbonate buffer (pH 9.6) was added to the mixture with steady stirring at room temperature for 16 h. After the reaction, the CTN-HRP solution with conjugation molar ratio as 29:1 was dialyzed with 0.01 M PBS for 2 days.

Crosslinking of CTN-HRP using CDI

The procedure of CTN-HRP conjugates with conjugation molar ratio as 29:1 via CDI was as following. Generally, 2 mg of CDI in 0.3 mL acetone was added to 1 mg of CTN in 0.3 mL acetone with continuous stirring for 3 h at 37°C. Then, 6 mg of HRP in 0.6 mL 0.1 M carbonate buffer (pH 9.6) was added to the reactant with steady stirring for 48 h at room temperature. After 48 h, the mixture was dialyzed by 0.01 M PBS for 2 days.

Coupling of CTN-HRP using 1,4-butanediol diglycidyl ether

The protocol of CTN-HRP conjugation with conjugation molar ratio as 29:1 via 1,4-Butanediol diglycidyl ether was as following. Briefly, 1 mg of CTN in 0.5 mL of sodium hydroxide solution (0.6 M) and 0.5 mL of sodium tetrahydridoborate solution (2 mg/mL) were mixed together with steady stirring at room temperature. Then, 0.5 μL of 1,4-butanediol diglycidyl ether solution was added to the reactant with continuous stirring at room temperature for 4 h. Next, 0.6 mL of HRP (6 mg in 0.1 M carbonate buffer, pH 9.6) solution was dropwise added to the mixture with stirring at 37°C for 24 h. Finally, 100 mg of glycine in 1 mL of deionized water was added to the reactant. After reacted for 48 h at 37°C, the mixture was dialyzed against 0.01 M PBS.

[a.] *A Graduate institute of Medicine, Chung Shan Medical University, Taichung 40201, Taiwan.*
[b.] *Graduate Institute of Toxicology, College of Medicine, National Taiwan University, Taipei 10051, Taiwan.*
[c.] *Department of Medical Research, Chung Shan Medical University Hospital, Taichung 40201, Taiwan.*
[d.] *Department of Biomedical Sciences, Chung Shan Medical University, Taichung 40201, Taiwan.*
* Correspondence: biingliu@ntu.edu.tw (B.-H.L.); fengyu@csmu.edu.tw (F.-Y.Y.);
Tel.: +886-2-23123456-88602 (B.-H.L.); +886-4-24730022-11816 (F.-Y.Y.)

Supplementary Figure

(A)

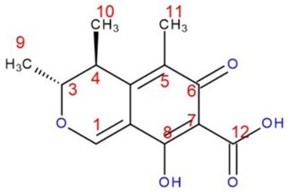

(B)

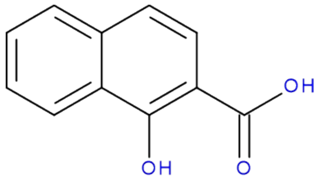

(C)

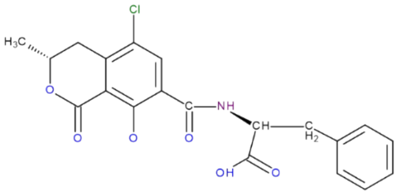

Fig S1. The chemical structure of (A) citrinin, (B) 1-Hydroxy-2-naphthoic acid and (C) Ochratoxin A.

(A)

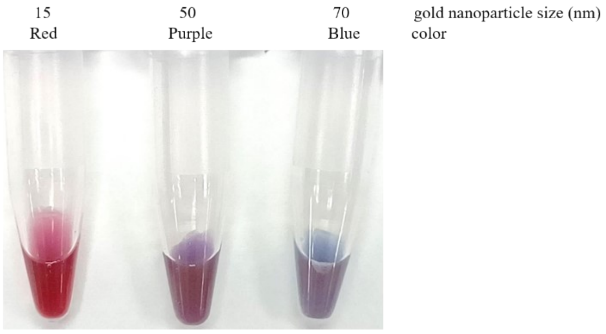

(B)

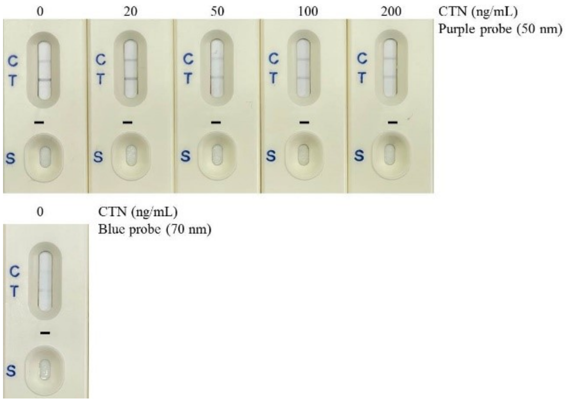

Fig S2. Comparison of gold nanoparticles with different sizes. (A) The CTN Ab were coupled to 15 nm (red), 50 nm (purple), and 70 nm (blue) nanoparticles. (B) The visual detection limit of the immunostrip using CTN Ab-GNPs probe with 50 nm and 70 nm nanoparticles.

This journal is © The Royal Society of Chemistry 20xx

Please do not adjust margins

Supplementary Table

Table S1. Validation of CTN ELISA kit analytical method with HPLC

		ELISA kit		Total
		Positive	Negative	
HPLC	Positive	2	1	3
	Negative	1	16	17

Specificity: $\frac{(Number\ of\ true\ negative)}{(Number\ of\ true\ negative + Number\ of\ false\ positve)} = \frac{16}{17}$

Sensitivity: $\frac{(Number\ of\ true\ positive)}{(Number\ of\ true\ positive + Number\ of\ false\ negative)} = \frac{2}{3}$

Table S2. Validation of CTN immunostrip screening method with HPLC

		Immunostrip		Total
		Positive	Negative	
HPLC	Positive	3	0	3
	Negative	4	13	17

Specificity: $\frac{(Number\ of\ true\ negative)}{(Number\ of\ true\ negative + Number\ of\ false\ positve)} = \frac{13}{17}$

Sensitivity: $\frac{(Number\ of\ true\ positive)}{(Number\ of\ true\ positive + Number\ of\ false\ negative)} = \frac{3}{3}$

This journal is © The Royal Society of Chemistry 20xx

Please do not adjust margins
